# Inference of differentially expressed genes using generalized linear mixed models in a pairwise fashion

**DOI:** 10.7717/peerj.15145

**Published:** 2023-04-03

**Authors:** Douglas Terra Machado, Otávio José Bernardes Brustolini, Yasmmin Côrtes Martins, Marco Antonio Grivet Mattoso Maia, Ana Tereza Ribeiro de Vasconcelos

**Affiliations:** 1 Laboratório de Bioinformática, Laboratório Nacional de Computação Científica, Petrópolis, Rio de Janeiro, Brazil; 2 Centro de Estudo em Telecomunicações, Pontifícia Universidade Católica do Rio de Janeiro, Rio de Janeiro, Brazil

**Keywords:** Differentially expressed genes, Random effects, Generalized linear mixed model, Preprocessing, Gene dispersion, DEGRE package

## Abstract

**Background:**

Technological advances involving RNA-Seq and Bioinformatics allow quantifying the transcriptional levels of genes in cells, tissues, and cell lines, permitting the identification of Differentially Expressed Genes (DEGs). DESeq2 and edgeR are well-established computational tools used for this purpose and they are based upon generalized linear models (GLMs) that consider only fixed effects in modeling. However, the inclusion of random effects reduces the risk of missing potential DEGs that may be essential in the context of the biological phenomenon under investigation. The generalized linear mixed models (GLMM) can be used to include both effects.

**Methods:**

We present DEGRE (Differentially Expressed Genes with Random Effects), a user-friendly tool capable of inferring DEGs where fixed and random effects on individuals are considered in the experimental design of RNA-Seq research. DEGRE preprocesses the raw matrices before fitting GLMMs on the genes and the derived regression coefficients are analyzed using the Wald statistical test. DEGRE offers the Benjamini-Hochberg or Bonferroni techniques for *P*-value adjustment.

**Results:**

The datasets used for DEGRE assessment were simulated with known identification of DEGs. These have fixed effects, and the random effects were estimated and inserted to measure the impact of experimental designs with high biological variability. For DEGs’ inference, preprocessing effectively prepares the data and retains overdispersed genes. The biological coefficient of variation is inferred from the counting matrices to assess variability before and after the preprocessing. The DEGRE is computationally validated through its performance by the simulation of counting matrices, which have biological variability related to fixed and random effects. DEGRE also provides improved assessment measures for detecting DEGs in cases with higher biological variability. We show that the preprocessing established here effectively removes technical variation from those matrices. This tool also detects new potential candidate DEGs in the transcriptome data of patients with bipolar disorder, presenting a promising tool to detect more relevant genes.

**Conclusions:**

DEGRE provides data preprocessing and applies GLMMs for DEGs’ inference. The preprocessing allows efficient remotion of genes that could impact the inference. Also, the computational and biological validation of DEGRE has shown to be promising in identifying possible DEGs in experiments derived from complex experimental designs. This tool may help handle random effects on individuals in the inference of DEGs and presents a potential for discovering new interesting DEGs for further biological investigation.

## Introduction

Sequencing technologies and bioinformatics tools allow the measurement of RNA transcripts in biological samples and the identification of DEGs in these samples (Costa-Silva, Domingues & Lopes, 2017; Wang, Gerstein & Snyder, 2009; Stark, Grzelak & Hadfield, 2019; Mao et al., 2020). DEGs are genes whose read counts are statistically different between two or more experimental conditions. The discovery of DEGs helps in answering several biological questions, some of which involve the functioning of an organism’s physiology (Yang et al., 2016; Tello-Ruiz, Jaiswal & Ware, 2022; Stelpflug et al., 2016), the detection of neoplastic tissue or other pathologies (Tang et al., 2018; Elemam et al., 2020), and the study of the microbiome (Niu et al., 2018), to name a few.

RNA-Seq experiments may have many variability sources, one with fixed effects and others that vary significantly among individuals. In experimental design, stable or fixed effects are more prevalent. On the other hand, random effects are more difficult to establish in the experiment’s configuration. Within-subject random effects are a powerful way of modeling individual variability (Penny et al., 2011; Gurka, Kelley & Edwards, 2012; Hedeker, Mermelstein & Demirtas, 2012). Widely used tools to infer DEGs, such as the DESeq2 (Love, Huber & Anders, 2014) and the edgeR (McCarthy, Chen & Smyth, 2012), do not consider random effects. The random effect in the model assumes that the explanatory variable has a relationship with the response variable across all observations, *i.e*., this effect may vary from one observation to another. For all observations, the random effects are modeled by probability distributions. A common approach is to analyze data with spatio-temporal autocorrelation with some variation in the observations (Nguyen & Nettleton, 2020). However, the random effects are not restricted to repeated measures in time or space. They could be related to variations across observations from other sources encountered in an individual (Gelman & Hill, 2006; Gomes, 2022; Hedeker, Mermelstein & Demirtas, 2012). For instance, individuals’ features among the groups may implicate the DEGs’ inference (Stevens et al., 2018). Moreover, treating random effects as fixed on the experimental design for DESeq2 and edgeR tools could increase potential DEGs’ false positive detection rate (Cui et al., 2016).

Here, we present DEGRE, a user-friendly computational tool that deploys generalized linear mixed models (GLMM) to assess DEGs using data preprocessing along with several metrics to evaluate performance, including false positive control. GLMMs are helpful in cases where there is both random variation within an experimental group and systematic differences among groups (Bolker et al., 2009). DEGRE is suitable for non-longitudinal experimental designs and proves to be promising for inferring DEGs in a pairwise fashion while accounting for fixed and random effects arising from individuals’ features.

## Materials and Methods

### Preprocessing of the count matrices

**Figure 1:**
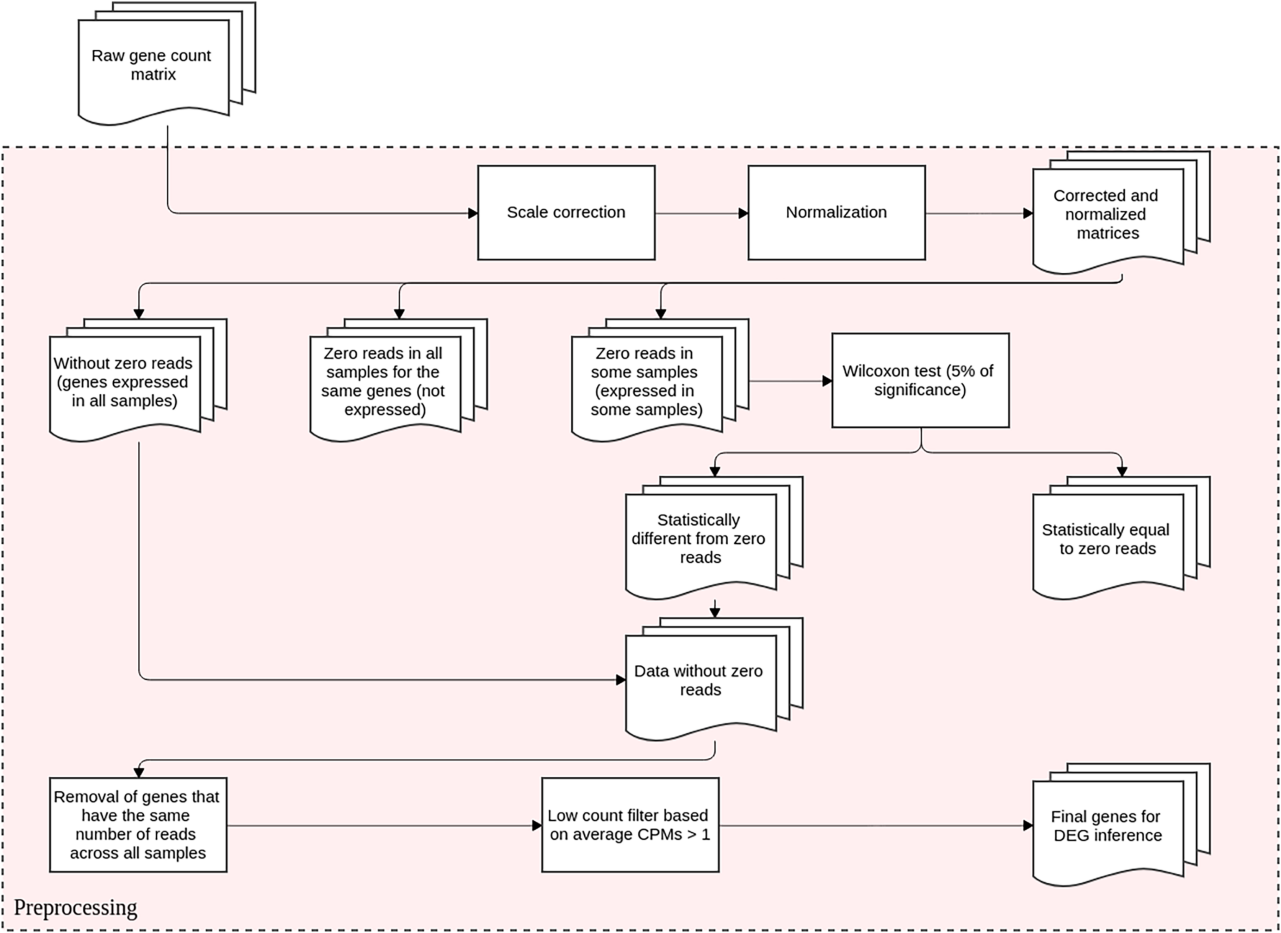
Preprocessing steps for RNA-Seq datasets before DEGs’ identification. The datasets are subjected to scale correction, followed by a normalization process. The resulting matrices are assessed based on gene expression. Genes with non-null counts are statistically evaluated for possible eliminations. The resulting dataset is directed to DEG inference.

This step is conducted before DEGs identification and it aims to reduce the technical variation in each experiment. It entails scale correction, matrix normalization, and filtering poorly expressed or non-expressed genes. The DEGs inference method proposed here is based upon differential expression analysis, that is, it models changes in gene expression levels across experimental conditions rather than simply measuring expression levels. The flowchart representation for this preprocessing step is displayed in Fig. 1.

We also used generalized linear models (GLMs) to validate the preprocessing step by comparing the DEGs detected to those produced by DESeq2 and edgeR. For that, the experimental design’s fixed effects were compared pairwisely, with the respective number of biological replicates for each experimental condition. The overdispersed genes, commonly modeled by GLM by means of negative binomial distribution with only fixed effects, were fitted using the MASS package (Venables & Ripley, 2002), version 7.3-53.1. The regression coefficients of the GLM derived for each gene were analyzed using the Wald test from the aod package (Lesnoff & Lancelot, 2012), version 1.3.1.

### Scale correction and normalization of the count matrices

Scale correction and normalization are preprocessing steps that reduce experimental technical bias and are commonly used in transcriptomic studies (Li et al., 2017, 2014). Thus, the matrices must contain the raw read counts rather than any prior normalizing ones. If the user adds the converted counts using any normalizing approach, the DEG inference will not accurately estimate the mean-variance relationship in the data, significantly impacting the statistical analysis. Denoting [*A*] as the count matrix with rows representing genes and columns representing samples, scale correction for each sequenced library begins by summing its column’s elements into a vector $\vec \nu = ({\nu _1},{\nu _2}, \ldots ,{\nu _n})$, where ${\nu _j} = \sum\nolimits_{i = 1}^n {{a_{ij}}} ,j \in \{ 1,...,n\}$ and ${a_{ij}}$ denotes entries in the matrix [*A*]. Next, its minimum element $\lambda$ is obtained, that is, $\lambda \buildrel \Delta \over = {\min _j}\;({\nu _j}).$

Next, we define the matrix $[{A^*}]$ with the same dimensions as [*A*], representing the corrected matrix. Its element in row $i$ and column $j$ is expressed by:

${[{A^*}]_{ij}} = a_{ij}^* = {\lambda \over {{v_j}}} \cdot {a_{ij}}\;,\;\;\;\forall \;\;i \in \{ 1,2,...,m\} \;\;{\rm{and}}\;\;j \in \{ 1,2,...,n\} .$

The relative log expression approach (Anders & Huber, 2010) is used to implement the normalizing step of matrix [A*]. Since this technique assumes that genes in the counting matrices are not differentially expressed, normalization aids in identifying those differentially expressed in a pairwise fashion.

### Filtering of unexpressed and low-expressed genes

Genes with zero reads in all biological replicates are eliminated. To identify non-expressed genes, genes that also have null counts are compared across the replicates using Wilcoxon’s nonparametric hypothesis test with a 5% significance level. For this purpose, the stats package, version 3.6.2, from the R programming language (R Core Team, 2022), version 4.0.4 is used. Genes not significantly different from null counts are eliminated from the analysis, and all the remaining are grouped with genes expressed in all replicates.

Genes with the same number of reads across all biological replicates in all conditions are also deleted since they are not differentially expressed. The dataset is then filtered for genes with low counts by means of the gene’s average count per million (CPM). Those genes with values smaller or equal to one are removed because weakly expressed genes reduce the sensitivity techniques for DEGs detection (Sha, Phan & Wang, 2015). The final datasets from the preprocessing step are used for dispersion evaluation and to infer the biological coefficient of variation (BCV).

To classify between equi- or overdispersed, the read counts of each gene along the biological replicates are modeled using the generalized linear model with the Poisson distribution. This model is estimated by means of the parglm package (Christoffersen, 2021), version 0.1.7, used in parallel mode, included in R programming language. Subsequently, Pearson’s chi-square test ratio is used to validate gene dispersion. The gene is classified as equidispersed if its value is less than or equal to one and overdispersed otherwise (Payne et al., 2018; Roback & Legler, 2021). The BCV inference is estimated for each gene before and after the preprocessing step using the *estimateCommonDisp* function from the edgeR package (Robinson, McCarthy & Smyth, 2010), version 3.32.1.

### DEGRE computational tool development

Primarily, DEGRE executes the preprocessing step previously described. Following this, a generalized linear mixed model (GLMM) with a negative binomial distribution is used to estimate the regression coefficients for inferring differentially expressed genes. For the GLMM fitting step in DEGRE, which employs both fixed and random effects, we used the glmmTMB package (Brooks et al., 2017), version 1.1.2.3. An example of the GLMM model (Stroup, 2012) is given below

$g(\mu |b) = X\beta + Zb,$

where (i) $g( \cdot )$ is a link function, (ii) $\mu |b$ is the conditional expectation of the observations given $b$, (iii) *X* is the design matrix for fixed effects, (iv) $\beta$ is the regression coefficients vector for fixed effects, (v) *Z* is the design matrix for random effects, and (vi) $b$ is the random effects’ vector.

The regression coefficients of the models derived for each gene are analyzed using the Wald test (Wald, 1943), also from the glmmTMB package. The *P*-value attributed to each gene must be adjusted by Bonferroni (Dunn, 1961) or Benjamini-Hochberg (Benjamini & Hochberg, 1995) techniques. The final dataset identifying DEGs and non-DEGs from the previous analysis is used to calculate (i) log2 fold-change values, by means of which a gene is classified as up- or downregulated and (ii) average logCPM. DEGRE does not define a specific value for the log2 fold-change cutoff and the user can select the desired cutoff value.

### Simulations of gene read count matrices in RNA-Seq

We created simulated count matrices of gene reads in which DEGs were known. The matrix rows represent the genes, while the matrix columns represent the biological replicates for two experimental conditions. These matrices were simulated with raw read counts for 38,293 genes in 2, 3, 4, 5, 6, 7, 8, 9, 10, and 15 biological replicates (using the “numberReps” parameter) in the mosim function from the MOSim package (Martínez-Mira, Conesa & Tarazona, 2018), version 1.4.0. The “times” parameter was set to zero corresponding to a non-longitudinal dataset and the “omics” parameter was defined as RNA-Seq. These numbers of replicates were chosen since they are commonly employed in differential expression analysis (Schurch et al., 2016). We investigated DEGs proportions ranging by 10%, 20%, and 30% for each matrix by setting the “diffGenes” parameter also in the mosim function. Moreover, for each matrix, the ratio of upregulated to downregulated genes is 1:1.

### Estimating the random effects for the simulated count matrices

We modeled the random effects using a variant of the normal probability distribution whose mean was estimated using public data in the Gene Expression Omnibus (GEO) database (www.ncbi.nlm.nih.gov/gds). The transcriptome studies chosen may have the same number of biological replicates as the previously described simulated datasets to prevent any bias in the mean parameter. The increased number of biological replicates might introduce additional biological variability into the datasets. Table 1 lists the transcriptome data identifiers (GEO IDs) utilized for random effects estimation.

**Table 1:** Real data was selected to be used in the estimation of the random effects.

GEO ID	Median number of reads	Number of genes before and after preprocessing	Replicates of the GEO data	Replicates of this work
GSE166276	20,154,892	28,930–12,669	2	2
GSE124993	41,509,271	63,925–14,728	3	3
GSE153744	44,875,441	26,265–14,193	4	4
GSE153823	18,307,656	60,606–14,739	5	5, 6, 7, 8, 9
GSE148892	3,035,785	63,772–13,731	12	10, 15

To simulate random effects, the raw data of each GEO is submitted to the preprocessing step (Additional File 1: Fig. S1). In order to determine read differences between the experimental conditions, the gene count average along the biological replicates is calculated in each transcriptome data for the two experimental conditions. The average differences along the experimental conditions are calculated and its average is used to estimate the mean parameter under the assumption of normal distribution. Seven values for the random effect standard deviations were selected, namely 100, 300, 600, 900, 1,200, 2,000, and 3,000, to cover a wide range of variability situations. The normal distributions simulated were then added to matrices with only fixed effects. At this step, if the random effect reduces gene reads, decreasing it to less than zero, the count value is replaced by zero. All gene reads were rounded to the nearest integer. The raw matrices generated with fixed and random effects were then the input for DESeq2, edgeR, and DEGRE.

### Evaluation of the identification of DEGs by the computational tools

The simulation of the count matrices is the basis for determining which genes are potential DEGs. This inference may be evaluated using recall, precision, and accuracy metrics.

### Public RNA-Seq data from patients with bipolar disorder for evaluation of the DEGRE computational tool

For the biological validation of DEGRE, we selected the dataset coded GSE80336 (GEO ID) that comprises RNA expression of brain samples of healthy people (*n* = 18) and patients with BD (*n* = 18). The corresponding read count matrix was submitted to the BCV estimation before and after the preprocessing step. The results found by DEGRE were compared to those generated by DESeq2 in a previous study (Pacifico & Davis, 2017). We used 5% of the FDR and Benjamini-Hochberg *P*-value correction for this comparison. Here, we used disease *vs*. control as the fixed effect and gender as the random effect (Thoral et al., 2021; Donovan et al., 2020; Kleyheeg et al., 2018) since studies indicate that gender can impact gene expression in transcriptomic analyses (Oliva et al., 2020), even if they are samples from the brain (Raznahan & Disteche, 2021). For Gene Ontology (GO) analysis, we used Ensembl IDs as input for the function basicProfile set to level 4, from the goProfiles R package (Sanchez, Ocana & Salicru, 2022), version 1.58.0.

## Results

### Filtering of the genes in the preprocessing step

The preprocessing step is assessed according to the filtering of (i) non-expressed genes, (ii) genes expressed in some biological replicates but with zero read counts in others, (iii) genes with equal read counts among biological replicates, and (iv) genes with low counts, in this order. We considered two scenarios: the first involves simulated datasets with only fixed effects, and the second considers simulated datasets with both fixed and random effects.

#### Scenario 1: Count matrices of RNA-Seq read counts only with fixed effects

**Figure 2:**
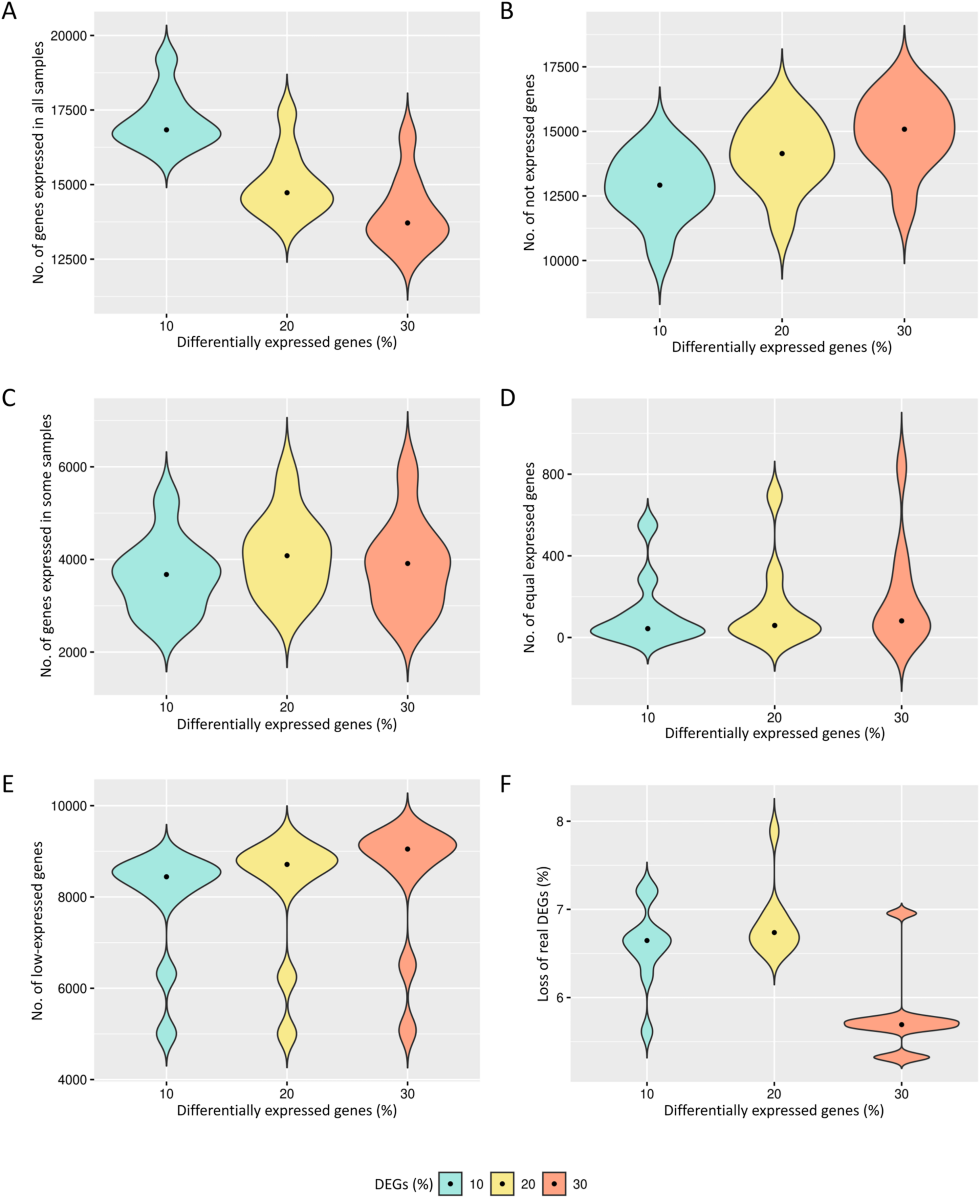
Results of the number of genes with features found in each data preprocessing step. The number of genes in matrices with fixed effects varied from (A) expressed in all samples, (B) not expressed, (C) expressed in some samples, (D) genes with equivalent expression across samples, (E) genes with low-expression, and (F) loss of DEGs (%).

The preprocessing selects genes that have reads in all biological replicates. The median number of genes expressed in all biological replicates are respectively 16,434, 14,163 and 13,092 for the cases of 10%, 20% and 30% of DEGs (Fig. 2A). All genes are kept in the datasets for further identification of low-expression genes.

The genes with zero read counts among the biological replicates are removed from the analysis (Fig. 2B). The median number of these genes are respectively 12,919, 14,141 and 15,083 for the cases of 10%, 20% and 30% of DEGs. The genes expressed in some biological replicates were also identified by the preprocessing (Fig. 2C). The median number of these genes were respectively 3,675, 4,080 and 3,912 for the cases of 10%, 20% and 30% of DEGs.

Genes with the same expression across biological replicates are removed from the analysis (Fig. 2D). The median of gene percentages that are not differentially expressed differed respectively 43, 59 and 81 for the cases of 10%, 20% and 30% of DEGs. Low-expressed genes were also identified (Fig. 2E), and their detection is known to be capable of improving the inference of DEGs. The median number of the low-expressed genes were respectively 8,442, 8,713 and 9,049 for the cases of 10%, 20% and 30% of DEGs. The loss of real DEGs during preprocessing can be considered low since its observed median rate has not exceeded 6.7% (Fig. 2F). The median proportion of these lost genes were 6.6%, 6.7%, and 5.7%, respectively, related to the total number of genes in matrices with 10%, 20% and 30% of DEGs.

#### Scenario 2: Count matrices of RNA-Seq read counts with the insertion of random effects

**Figure 3:**
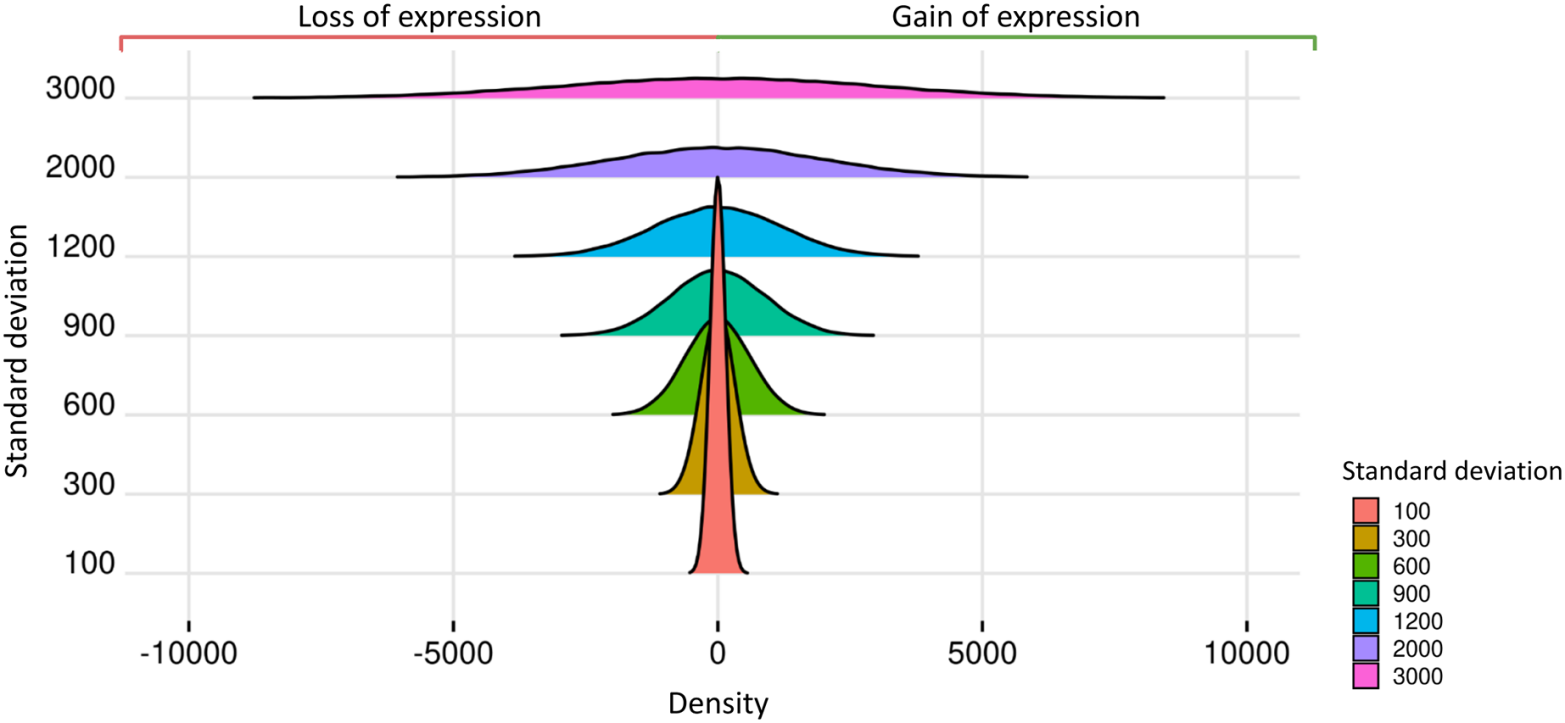
Normal distributions associated with random effects.

Prior datasets from scenario 1 were subjected to the insertion of random effects. The predicted impact of these effects on gene expression is related to the Standard Deviations of the Normal Distribution (SDND), which were selected as the vector *t* = 100, 300, 600, 900, 1,200, 2,000, 3,000. The mean parameter for the estimation of the random effects was 2.028 in matrices with two replicates, 9.937 for three replicates, 7.158 for four replicates, −3.429 for five to nine replicates, and −1.615 for ten and fifteen replicates. We present in Fig. 3 the expected impact of adding a simulated normal distribution varying standard deviation with the values on $t$ and with zero mean. The higher the random effect’s standard deviation, the more significant its deleterious impact on the datasets.

The results reveal that greater SDNDs reduce the number of genes expressed in all biological replicates (Additional File 1: Fig. S2A). Also, no gene is completely unexpressed, resulting in high gene expression variation across the transcriptome (Additional File 1: Fig. S2B). In the context of gene expression among biological replicates, the median number of expressed genes is reduced, meaning that gene expression was absent in some replicates (Additional File 1: Fig. S2C). In addition, the number of genes equally expressed in all replicates decreases (Additional File 1: Fig. S2D). Interestingly, for the case of SDND 100, we detected low-expressed genes, indicating greater heterogeneity among biological replicates (Additional File 1: Fig. S2E). For each SDND, the DEGs’ loss was insignificant, thus implying that the preprocessing step filters genes without affecting true DEGs removal (Additional File 1: Fig. S2F).

The increase in the observed DEGs proportion exhibits the same pattern described before. Additional File 1, Figs. S3 and S4, respectively, show gene filters for datasets containing 20% and 30% of DEGs. Filtering has produced comparable results for the three DEG proportions, although the number of expressed genes in all replicates has decreased for matrices with 20% and 30% of DEGs compared to 10%. The loss of DEGs was equivalent for the three cases, suggesting that preprocessing has removed genes that might interfere with the subsequent inference of DEGs.

### Measuring the effect of the preprocessing step on gene dispersion

Under previously mentioned scenarios, the preprocessing step was used to prepare the data for DEGs’ inference.

#### Scenario 1: Count matrices of RNA-Seq read counts only with the fixed effects

**Figure 4:**
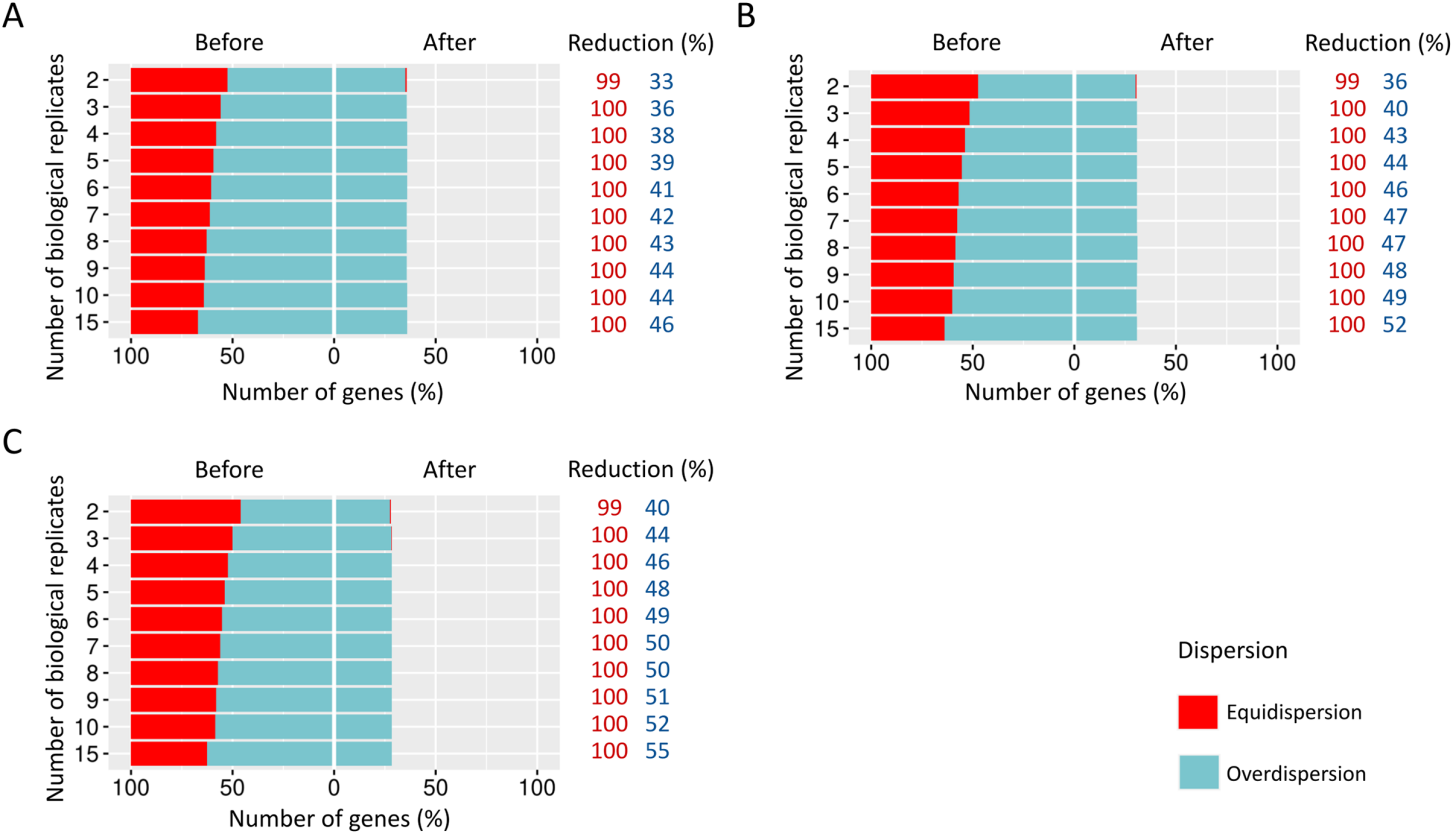
Number of equidispersed (in red) and overdispersed (in blue) genes in the counting matrices containing (A) 10%, (B) 20%, and (C) 30% of DEGs before and after the preprocessing step. The reduction percentage for each result refers to the number of equidispersed and overdispersed genes before and after the preprocessing application in matrices with fixed effects.

We estimated the dispersion of each gene and then examined how it is influenced before and after the preprocessing step (Fig. 4).

The proportions corresponding to datasets with 10% (Fig. 4A), 20% (Fig. 4B), and 30% (Fig. 4C) of DEGs have a considerable amount (33–54%) of equidispersed genes before the preprocessing step. After the preprocessing, the number of equidispersed genes was practically null. Therefore, the preprocessing has preserved the number of overdispersed genes, which is useful in properly predicting DEGs.

#### Scenario 2: Count matrices of RNA-Seq read counts with the insertion of random effects

**Figure 5:**
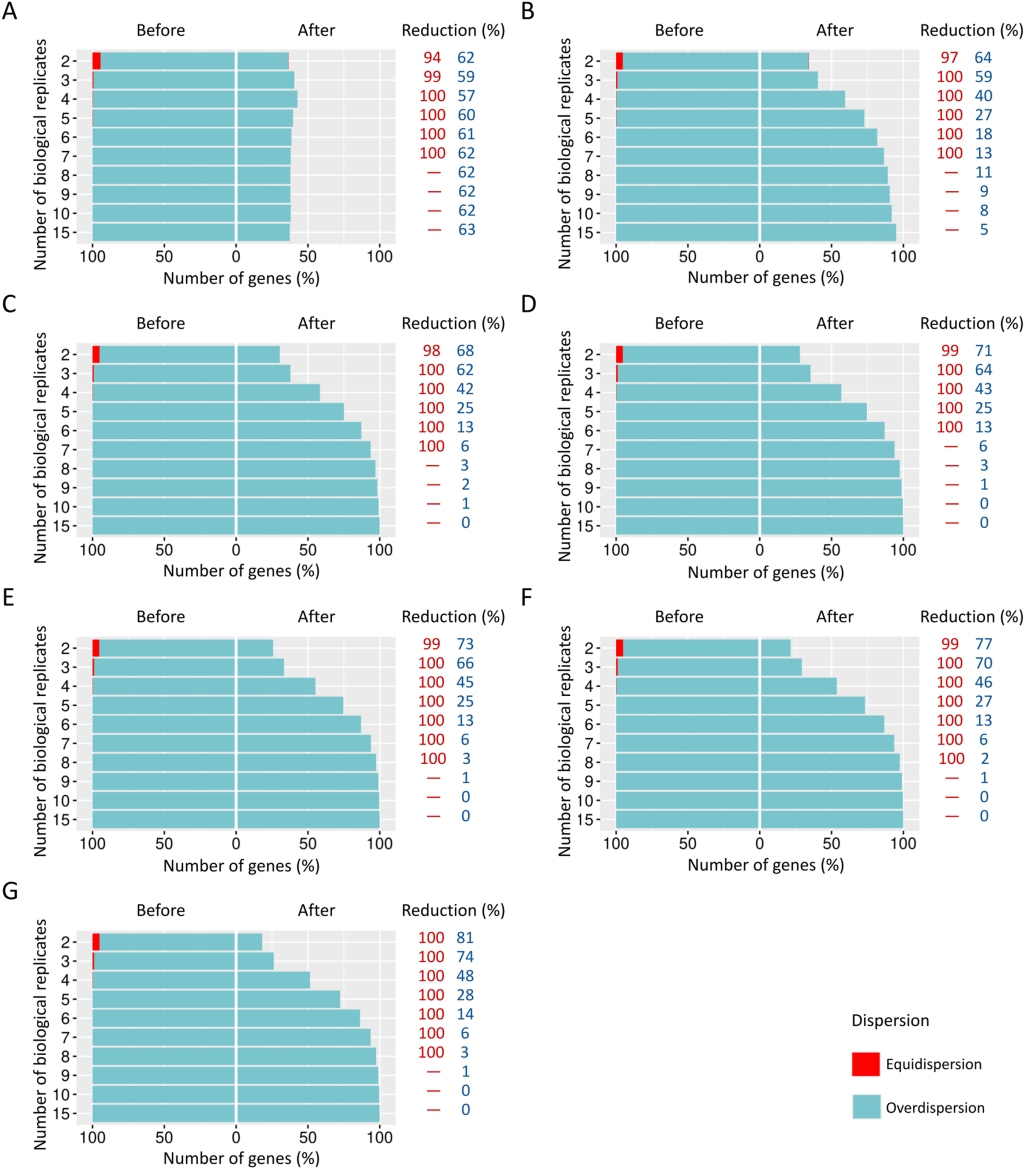
Number of equidispersed (in red) and overdispersed (in blue) genes in the counting matrices with 10% of DEGs before and after the preprocessing step. The SDNDs vary between (A) 100, (B) 300, (C) 600, (D) 900, (E) 1,200, (F) 2,000, and (G) 3,000. The percentage reduction for each result refers to the number of equidispersed and overdispersed genes before and after the preprocessing application in matrices with fixed and random effects.

When SDND is 100, a substantial and a moderate decrease is respectively observed on equidispersed and overdispersed genes in all biological replicates (Fig. 5A). However, SDND 300 (Fig. 5B), 600 (Fig. 5C), 900 (Fig. 5D), 1,200 (Fig. 5E), 2,000 (Fig. 5F), and 3,000 (Fig. 5G) has resulted in a 100% reduction in fewer biological replicates for all equidispersed genes. A similar profile was found in datasets with 20% of DEGs (Additional File 1: Fig. S5) and 30% of DEGs (Additional File 1: Fig. S6).

### The preprocessing step in modeling biological variability arising from random effects

**Figure 6:**
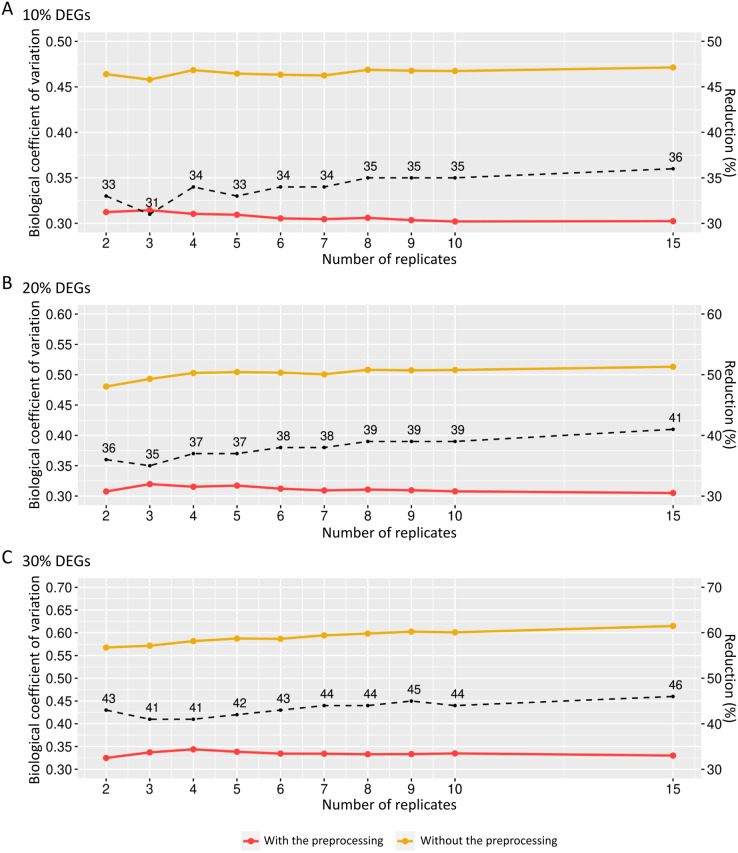
Values for the biological coefficient of variation in matrices with only fixed effects before (in yellow) and after (in red) the preprocessing step. (A), (B) and (C) respectively represent the coefficients for the cases of 10%, 20% and 30% of DEGs. The percentage of the coefficient reduction before and after the preprocessing step for each replicate is indicated by the black dashed line.

The preprocessing’s influence on biological variability was first inferred using matrices with fixed effects (Fig. 6). The biological variation in the replicates before preprocessing on matrices containing 10% of DEGs ranges from 0.46 and 0.47 (Fig. 6A). When the preprocessing step was applied, the variation was between 0.30 and 0.31. Similarly, the decreasing proportion occurs in Fig. 6B, where the BCV before and after the preprocessing step ranges, respectively, 0.48–0.51 and 0.30–0.32. On the other hand, Fig. 6C shows that the decrease rate before and after the data preprocessing step is higher than the corresponding ones in Figs. 6A and 6B. The BCV before the preprocessing step ranged from 0.57 to 0.61, while after the preprocessing, it ranged from 0.32 to 0.34.

**Figure 7:**
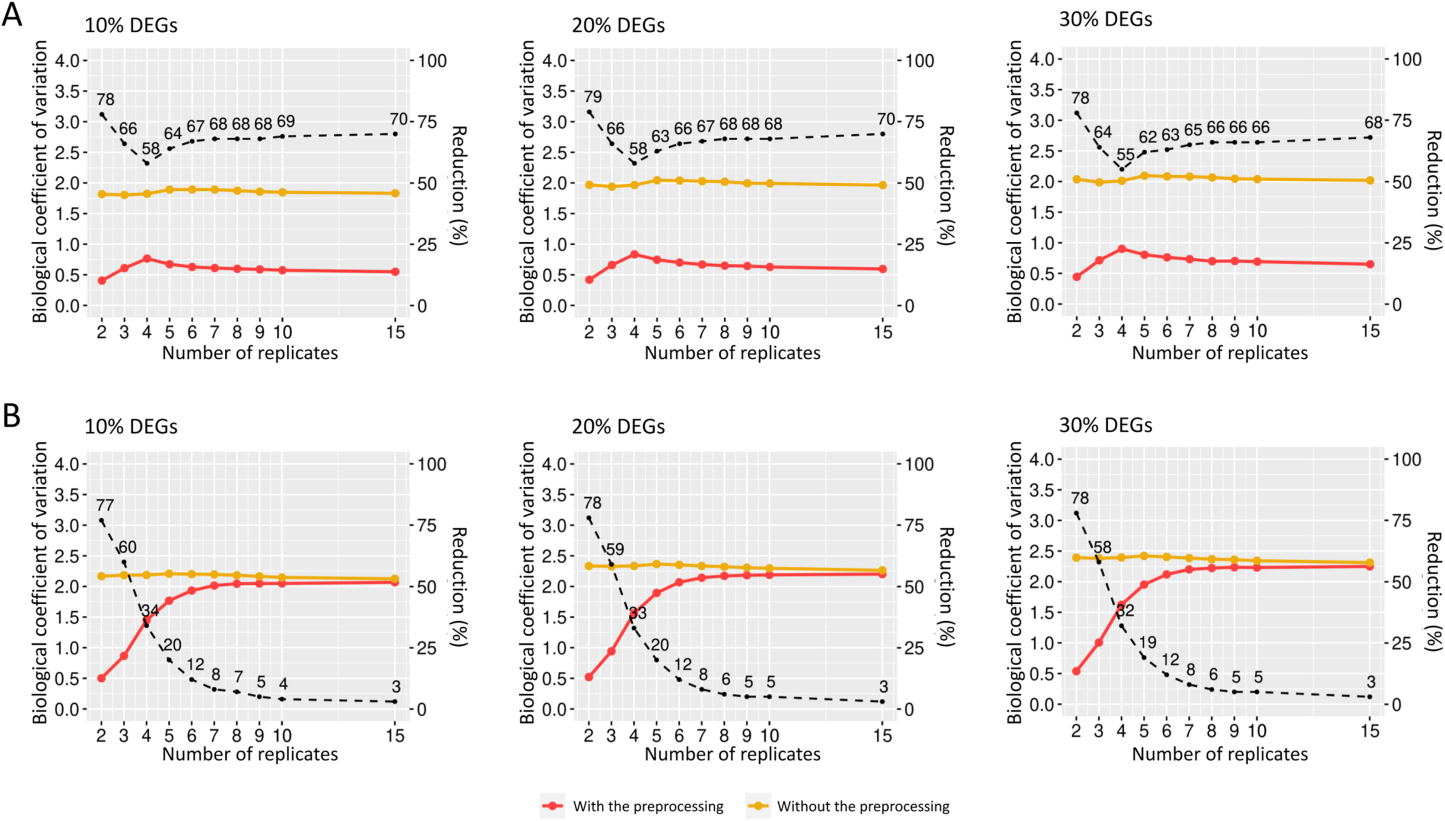
Biological coefficient of variation for estimated random effects datasets with SDND 100 (A) and SDND 300 (B). The black dashed line indicates the percentage of BCV decrease before and after the preprocessing phase for each point on the graph.

We also examined how the BCV changes in datasets with added random effects. For the case of SDND 100 (Fig. 7A), there is a decrease in BCV in matrices with 10%, 20%, and 30% of DEGs. There were no significant differences in BCV between the three DEG proportions. SDND 300 results in a BCV increase before the preprocessing step (Fig. 7B), and they are shown below for matrices with 10%, 20%, and 30% of DEGs. For the other SDNDs studied in this work the BCV increase presents the same pattern.

### Evaluation of the preprocessing step in identifying differentially expressed genes using the generalized linear models

**Figure 8:**
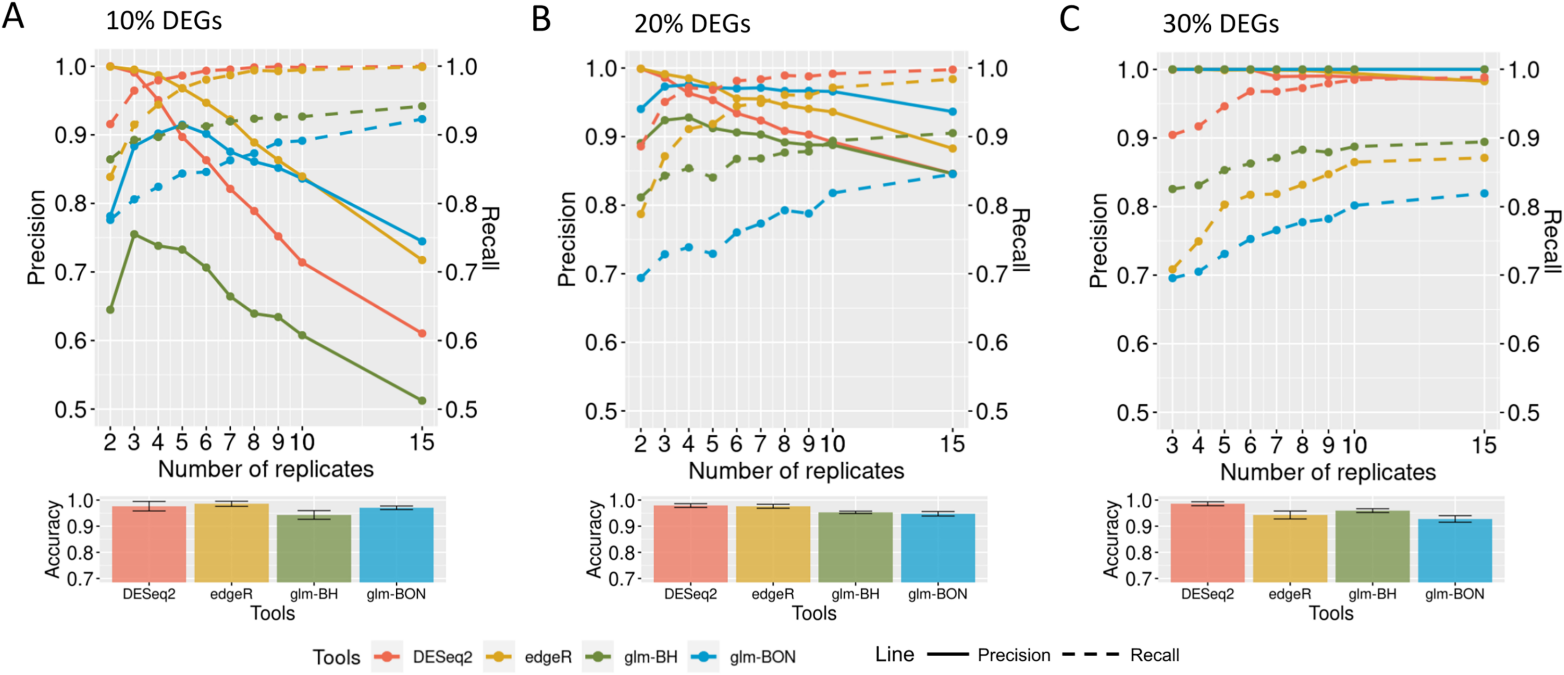
Evaluation of DEGs identification on simulated datasets using GLM models. (A), (B) and (C) correspond to the datasets with 10%, 20%, and 30% of DEGs, respectively. The solid line is the precision rate, and the dashed line is the recall rate. Below each figure is shown the accuracy’s mean and standard deviation. The computational tools are DESeq2, and edgeR and the models are the GLMs with the Bonferroni (BON) or Benjamini-Hochberg (BH) *P*-value correction.

To evaluate how the preprocessing might affect the identification of DEGs, fixed effects count matrices were submitted to GLMs with Benjamini Hochberg (glm-BH) or Bonferroni (glm-BON) *P*-value adjustments. In datasets with 10% of DEGs, the increasing number of biological replicates submitted to all tools resulted in a precision decrease (Fig. 8A), highlighting the fact that all tools presented a high percentage of false positives. However, the recall has improved, indicating that the false negative rate has been reduced. The evaluation metrics in Fig. 8B show that all tools have improved their performance in datasets with 20% of DEGs. A similar result is observed in datasets containing 30% DEGs (Fig. 8C). Notice that the mean accuracies remain equivalent in all tools and DEG cases.

### Identification of the differentially expressed genes using the DEGRE tool

**Figure 9:**
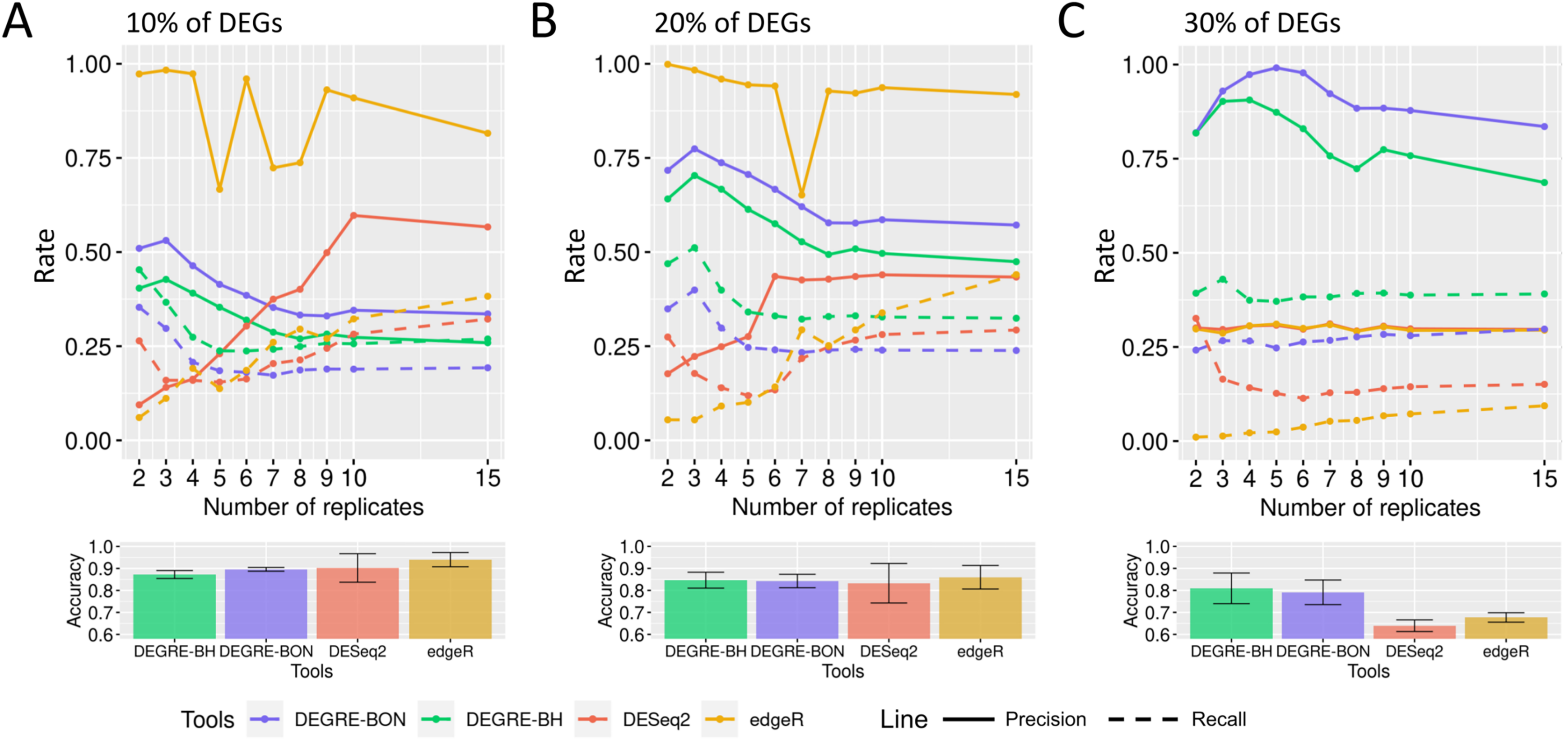
Identification of DEGs by the computational tools in datasets with random effects. (A), (B) and (C) are the datasets with 10%, 20%, and 30% of DEGs, respectively. The solid line is the precision rate, and the dashed line is the recall rate. Below each figure is the mean and standard deviation of the accuracy. The computational tools are DEGRE-BON, DEGRE-BH, DESeq2, and edgeR.

When evaluating the DEGRE performance of DEGs’ detection in matrices with the added random effects, those containing 10% of DEGs have shown precision and recall rates above those values observed in DESeq2 up to six biological replicates (Fig. 9A). The edgeR displayed the highest precision among all replicates. Still, its recall rate remained below the DEGRE with Benjamini Hochberg (DEGRE-BH) *P*-value correction, also up to six biological replicates. For matrices with 20% of DEGs, the DEGRE tool exhibits a high precision but below those observed in the edgeR case (Fig. 9B). However, we notice that DEGRE-BH kept its recall above the registered by the edgeR, indicating that the false negatives are more controlled by DEGRE-BH than by edgeR. For matrices with 30% of DEGs, we notice that precision and recall were above those presented by DESeq2 and edgeR, indicating that in datasets heavily impacted by random effects, DEGRE may help the detection of real DEGs (Fig. 9C). We highlight that the mean accuracy for 10% and 20% of DEGs cases are the same for all tools. However, for 30% of DEGs, the accuracy of the DEGRE-BH and DEGRE with Bonferroni (DEGRE-BON) *P*-value correction was above those measured on DESeq2 and edgeR tools.

### Identification of the differentially expressed genes in a case study using the DEGRE tool

Here, we examined a public dataset of RNA-Seq involving healthy individuals and patients with bipolar disorder (BD), showing a BCV of 0.37. After the preprocessing step, the BCV measured was 0.32, indicating that the preprocessing has reduced the technical variation in this experiment. Of the total 47,886 genes, 29,734 were deleted, resulting in a dataset of 18,152 genes for further DEGs’ discovery. DEGRE identified 133 genes as differentially expressed candidates (Additional File 2).

**Figure 10:**
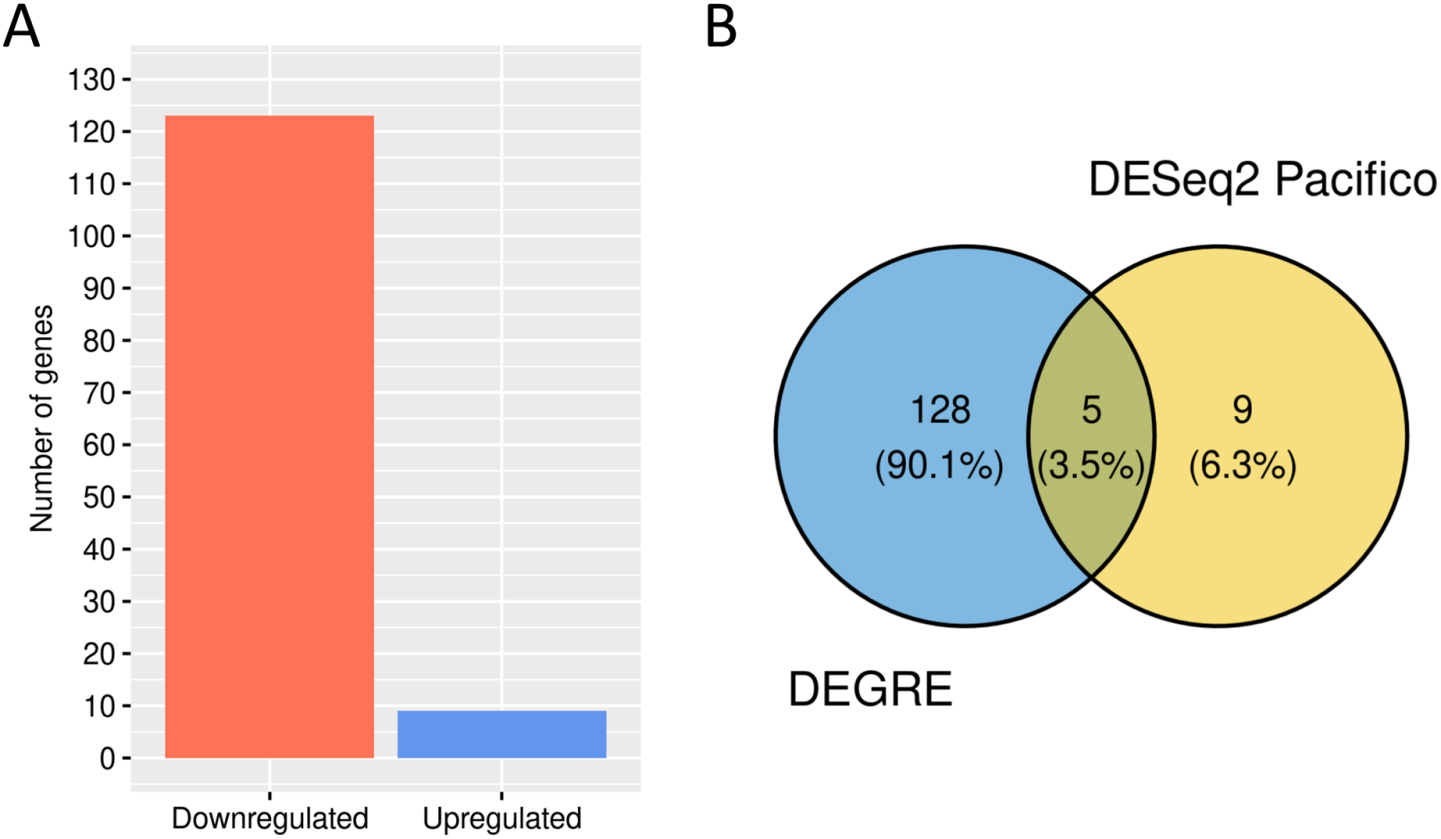
Number of genes identified as differentially expressed by DEGRE. (A) 124 genes were identified as downregulated, and nine were identified as upregulated. (B) The intersection of the 133 genes identified by DEGRE with the findings of Pacifico & Davis (2017).

We performed a GO analysis to identify the biological processes in which these 133 potential DEGs might be involved (Additional File 3). DEGRE has detected genes related to metabolic processes, with a higher frequency number in the macromolecule metabolic process (GO:0043170, *n* = 58) and also in the immune response, with a higher frequency number in the regulation of the immune system process (GO:0002682, *n* = 11). When the DEGRE tool was compared with the DESeq2 used in the previous study (Pacifico & Davis, 2017), we identified 128 different gene candidates that were differentially expressed but not noticed by the above reference (Fig. 10).

The nine genes not identified by the DEGRE tool but identified by Pacifico & Davis (2017) have Q-values above the 5% FDR. The 128 genes only identified by DEGRE as possible DEGs have an ontology that relates them to biological processes involved in patients with BD (Beech et al., 2010). An association with BD in 20.31% (*n* = 26) of the genes has been identified in our study (Additional File 4). Of these, the genes *EIF4EBP1*, *SLC5A3*, *CYP7B1*, and *AHCTF1* are here discussed as examples of our method’s capability in discovering potential candidate genes linked to BD.

The *EIF4EBP1* gene encodes a translation repressor protein that is the major substrate of the mTOR signaling pathway (Akbarian et al., 2020; Cha et al., 2015). This gene was found to be downregulated in patients with bipolarity (log2 fold-change = −1.15). The *SLC5A3* gene is also downregulated (log2 fold-change = −1.05) and encodes the protein sodium-coupled myo-inositol transporter 1, which is related to the transport of myo-inositols for the cell (Schneider, 2015; Dai et al., 2016). The *CYP7B1* gene encodes a member protein of the cytochrome P450 superfamily of enzymes (Chiang, 2004). These enzymes have functions related to the metabolism of oxysterols and steroid hormones, including neurosteroids (Chiang, 2004). DEGRE classifies the *CYP7B1* gene expression profile as upregulated in patients with bipolar disorder compared to healthy individuals (log2 fold-change = 0.24). The *AHCTF1* gene encodes the nucleoporin ELYS that regulates the nucleus size in mammalian cells by controlling the number of nuclear pore complexes and the ability to import molecules into the nucleus (Jevtić et al., 2019). The *AHCTF1* gene was downregulated in patients with bipolarity compared to healthy individuals (log2 fold-change = −0.22).

## Discussion

Here, we discuss the DEGRE, a user-friendly tool. This R package has a new way of preprocessing RNA-Seq counting matrices before inferring putative DEGs using generalized linear mixed models in a pairwise fashion. DEGRE allows users to consider random and fixed effects in the modeling. We compared the DEGs’ inference performance with those produced by DESeq2 and edgeR tools by means of the accuracy, precision, and recall metrics. We simulated count matrices with gene reads that ranged between 10%, 20%, and 30% of DEGs in 2, 3, 4, 5, 6, 7, 8, 9, 10, and 15 biological replicates. MOSim package’s simulation creates replicates for each experimental condition and they are treated as biological variations since the technical variation is uniformly present throughout all libraries. Preprocessing showed the absence of gene transcription in the biological replicates, meaning that these genes do not produce RNA, which is required for the organism’s physiology (Fernandes et al., 2019; Sabari et al., 2017). Many genes had different numbers of reads in all replicates, thus demonstrating the replicates biological diversity. Poorly expressed genes were removed from the analysis as they may limit the sensitivity for detecting potential DEGs (Sha, Phan & Wang, 2015).

The BCV was inferred to assess how preprocessing reduces technical variability in the counting matrices in order to keep it better controlled. The BCV values remained above 0.45 in all biological replicates under all three DEGs’ proportions. However, after performing the preprocessing step, the BCVs decreased to values below 0.35, indicating that this step removes the technical variability and preserves the biological variability in each dataset.

The preprocessing step with posterior DEGs’ inference using GLM in matrices with only fixed effects performs similarly to those observed in DESeq2 and edgeR. In matrices with 10% of DEGs, the increase in the number of biological replicates reduced the precision metric for all tools. It indicates that the increased biological variability induced by the number of replicates has also increased the false positive rate. On the other hand, the increase in recall illustrates that the false negative rate is better controlled. The comparative results with the remaining DEGs’ proportions indicate that the development of the preprocessing step has identified and removed genes that could subsequently impair the statistical modeling for identifying DEGs.

Random effects are simulated using the nonnegative-integer truncated normal distribution, and its mean parameter was estimated using real transcriptome data. The standard deviation parameter ranges in the set 100, 300, 600, 900, 1,200, 2,000, and 3,000, and consequently the random effects’ variability ranged from low to high, impacting the loss and gain of gene expression. Although random effects on datasets lowered gene read counts in some biological replicates, no gene is completely unexpressed since the genes could gain expression in the others replicates.

The amount of equidispersed and overdispersed genes was measured before and after the preprocessing step. The addition of random effects reduced the number of overdispersed genes for the case where SDND is equal to 100. However, for larger SDNDs, the number of overdispersed genes has increased. This increase was visualized for the three DEGs proportions considered, suggesting that the DEGs increase does not influence the amount of equi and overdispersed genes.

While the BCV remained around 0.30 in datasets with only fixed effects, in those with also random effects the BCV for SDND 100 after preprocessing was above 0.50. For SDNDs higher than 100, BCVs are above 1.50, indicating that there is high variability from random effects to be modeled with DEGRE.

For the 10% case, DEGRE has kept precision and recall above those observed on DESeq2 up to six biological replicates indicating fewer false negatives and false positives than in the DESeq2 case. It has already been shown that if the DESeq2 treats random effects as fixed, it could produce a high rate of false positives (Cui et al., 2016), corroborating the results of this study. Compared to edgeR, DEGRE displays a higher recall rate up to six biological replicates indicating that DEGRE controls false negatives better, *i.e*., DEGRE is not attributing as non-DEGs the genes that are really DEGs.

For the 20% case, DEGRE maintains its recall higher than those observed on edgeR up to nine biological replicates. Although the edgeR’s associated precision is higher, its lowest recall value indicates that DEGRE identifies more real DEGs. We expect more genes differentially expressed with increased biological variability due to the random effects. For that, we used matrices with 30% of DEGs to assess how random effects would influence the DEGs identification under all the tools. DEGRE controls the random effects better, thus maintaining precision and recall above those levels associated with DESeq2 and edgeR. Hence DEGRE has the potential for better control of biological variability when inferring DEGs under fixed and random effects.

We analyzed genes identified by DEGRE in a transcriptomic study involving patients with BD. We detected that the DEGs inferred have biological processes already described for this disorder, such as those related to metabolism and the immune system (SayuriYamagata et al., 2017; Barbosa et al., 2014; Rosenblat & McIntyre, 2017), thus evidencing that DEGRE identified potential DEGs candidates for further analysis. From those DEGs identified, we highlight the findings of *EIF4EBP1*, *SLC5A3*, *CYP7B1*, and *AHCTF1*. The *EIF4EBP1* and *SLC5A3* genes are described as being related to bipolar disorder since *EIF4EBP1* is involved in mTOR signaling that is altered in patients with bipolarity (Park et al., 2022; Vanderplow et al., 2021) and *SLC5A3* is the focus of current research regarding inositol transport system in those patients (Vawter et al., 2019; Yu & Greenberg, 2016; Silverstone, McGrath & Kim, 2005; Van Calker & Belmaker, 2000).

In addition, the *CYP7B1* gene is related to cholesterol metabolism. Using blood plasma as biological material, patients with BD have a lower concentration of hydroxycholesterol when compared to healthy individuals (Guidara et al., 2021). This result is related to the expression profile identified by the DEGRE tool, which has classified this gene as upregulated in patients with BD compared to healthy individuals. The impairment of cholesterol and oxysterol metabolism can affect the organism’s homeostasis through the cytotoxicity caused by oxidative stress, apoptosis, and synaptic dysfunction, and it has already been shown that this impairment has a direct implication in the pathophysiology of BD (Sun et al., 2017; Kim, Rapoport & Rao, 2010). Consequently, identifying the *CYP7B1* gene as a possible DEG does allow it to be one of the candidates to explain the molecular mechanisms in metabolic pathways that are possibly dysregulated in patients with BD.

Another example of consistent identification of possible DEGs is the *AHCTF1* gene. In a study to identify downregulated genes based on a transcriptomic meta-analysis in the peripheral blood of individuals with BD, the *AHCTF1* gene is identified as downregulated compared to healthy individuals (log2 fold-change = −0.17) (Hess et al., 2020). DEGRE also indicated that this gene is downregulated in patients with BD, showing that these results, although performed in brain tissues, are consistent with findings in the scientific literature.

We used the BD dataset as an application of how our method works in real datasets, and how the random effect’s inclusion could aid DEG’s inference *via* DEGRE. The inclusion of random effects can result in more data being investigated. Although DEGRE detected other DEGs that could be linked to bipolar disorder, a subsequent study would require a more in-depth biological analysis.

DEGRE proposes the implementation of a new preprocessing method and the inclusion of random effects in the experimental design proved to be sensitive in detecting true DEGs in matrices with high biological variability. This tool seems promising because it could infer potential DEGs identified in other reports in the scientific literature in patients with bipolar disorder.

## Conclusions

The DEGRE tool discussed here presents the potential for identifying Differentially Expressed Genes (DEGs) in experimental designs with fixed and random effects. DEGRE displays a new preprocessing procedure for count matrices of RNA-Seq data and uses Generalized Linear Mixed Models (GLMM) to infer DEGs. Although well-established tools infer DEGs using GLMs, DEGRE allows the insertion of random effects in its inference, thus contributing to identifying potential candidates for real differentially expressed genes. DEGRE is appropriate for non-longitudinal experiment designs, and future analyses including these types of experiments can be conducted.

Including random effects in the experimental design of the particular case study involving patients with bipolar disorder allowed DEGRE to identify more DEGs candidates. As reported in the scientific literature, biological processes related to these genes are associated with patients affected by bipolar disorder, and consequently, their analysis should be further pursued. This biological motivation allows us to consider DEGRE a promising tool for inferring true positive DEGs in RNA-Seq research.

## Supplemental Information

10.7717/peerj.15145/supp-1 Supplemental Information 1 Random effects’ estimation for the simulated count matrices. Public raw data are directed to preprocessing followed by replicate averages for the two experimental conditions and the average difference is calculated between them. For each average, the chosen set of standard deviations is used to generate normal distribution values to be added to the fixed effects’ matrices. Negative values are replaced by zero and the whole matrix values are rounded to the nearest integer. In this final step, the generated matrices with fixed and random effects are the input for DESeq2, edgeR and DEGRE.

10.7717/peerj.15145/supp-2 Supplemental Information 2 Results of the number of genes with features found in data preprocessing step in the case of 10% of DEGs. The number of genes in matrices with fixed and random effects varied from (A) expressed in all samples, (B) not expressed, (C) expressed in some samples, (D) genes with equivalent expression across samples, (E) genes with low-expression, and (F) loss of DEGs (%).

10.7717/peerj.15145/supp-3 Supplemental Information 3 Results of the number of genes with features found in data preprocessing step in the case of 20% of DEGs. The number of genes in matrices with fixed and random effects varied from (A) expressed in all samples, (B) not expressed, (C) expressed in some samples, (D) genes with equivalent expression across samples, (E) genes with low-expression, and (F) loss of DEGs (%).

10.7717/peerj.15145/supp-4 Supplemental Information 4 Results of the number of genes with features found in data preprocessing step in the case of 30% of DEGs. The number of genes in matrices with fixed and random effects varied from (A) expressed in all samples, (B) not expressed, (C) expressed in some samples, (D) genes with equivalent expression across samples, (E) genes with low-expression, and (F) loss of DEGs (%).

10.7717/peerj.15145/supp-5 Supplemental Information 5 Number of equidispersed (in red) and overdispersed (in blue) genes in the counting matrices containing 20% of DEGs before and after the preprocessing step. The SDNDs vary between (A) 100, (B) 300, (C) 600, (D) 900, (E) 1200, (F) 2000, and (G) 3000. The reduction percentage for each result refers to the number of equidispersed and overdispersed genes before and after the preprocessing application in matrices with fixed and random effects.

10.7717/peerj.15145/supp-6 Supplemental Information 6 Number of equidispersed (in red) and overdispersed (in blue) genes in the counting matrices containing 30% of DEGs before and after the preprocessing step. The SDNDs vary between (A) 100, (B) 300, (C) 600, (D) 900, (E) 1200, (F) 2000, and (G) 3000. The reduction percentage for each result refers to the number of equidispersed and overdispersed genes before and after the preprocessing application in matrices with fixed and random effects.

10.7717/peerj.15145/supp-7 Supplemental Information 7 The 133 genes identified by DEGRE as differentially expressed candidates. The identification of the genes, log2FC value, *P*-value, Q-value and average log CPM value.

10.7717/peerj.15145/supp-8 Supplemental Information 8 Results of gene ontology for the 133 genes identified by DEGRE as differentially expressed candidates. The biological process identification with the respective quantity of genes.

10.7717/peerj.15145/supp-9 Supplemental Information 9 Association of DEGs identified by DEGRE with bipolar disorder.

## Additional Information and Declarations

### Data Availability

The following information was supplied regarding data availability: The RNA-Seq datasets used in this study are available at the Gene Expression Omnibus repository: GSE166276, GSE124993, GSE153744, GSE153823, GSE148892, and GSE80336. The Supplemental Materials, a vignette, and the R package are available at Github and Zenodo: https://github.com/labinfo-lncc/DEGRE; Douglas Terra Machado, Otávio José Bernardes Brustolini, Yasmmin Côrtes Martins, Marco Antonio Grivet Mattoso Maia, & Ana Tereza Ribeiro de Vasconcelos. (2022). DEGRE: Inferring Differentially Expressed Genes using Generalized Linear Mixed Models (0.2.0). Zenodo. https://doi.org/10.5281/zenodo.7636564. The package is published by CRAN at: https://CRAN.R-project.org/package=DEGRE; Douglas Terra Machado, Otávio José Bernardes Brustolini, Yasmmin Côrtes Martins, Marco Antonio Grivet Mattoso Maia, & Ana Tereza Ribeiro de Vasconcelos. (2022). DEGRE: Inferring Differentially Expressed Genes using Generalized Linear Mixed Models (0.2.0). Zenodo. https://doi.org/10.5281/zenodo.7636564.
