# Supplementary figures and images for: Inference of differentially expressed genes using generalized linear mixed models in a pairwise fashion
Source: PeerJ. 2023 Apr 3;11:e15145. doi: 10.7717/peerj.15145 (PMC10078460; doi:10.7717/peerj.15145)

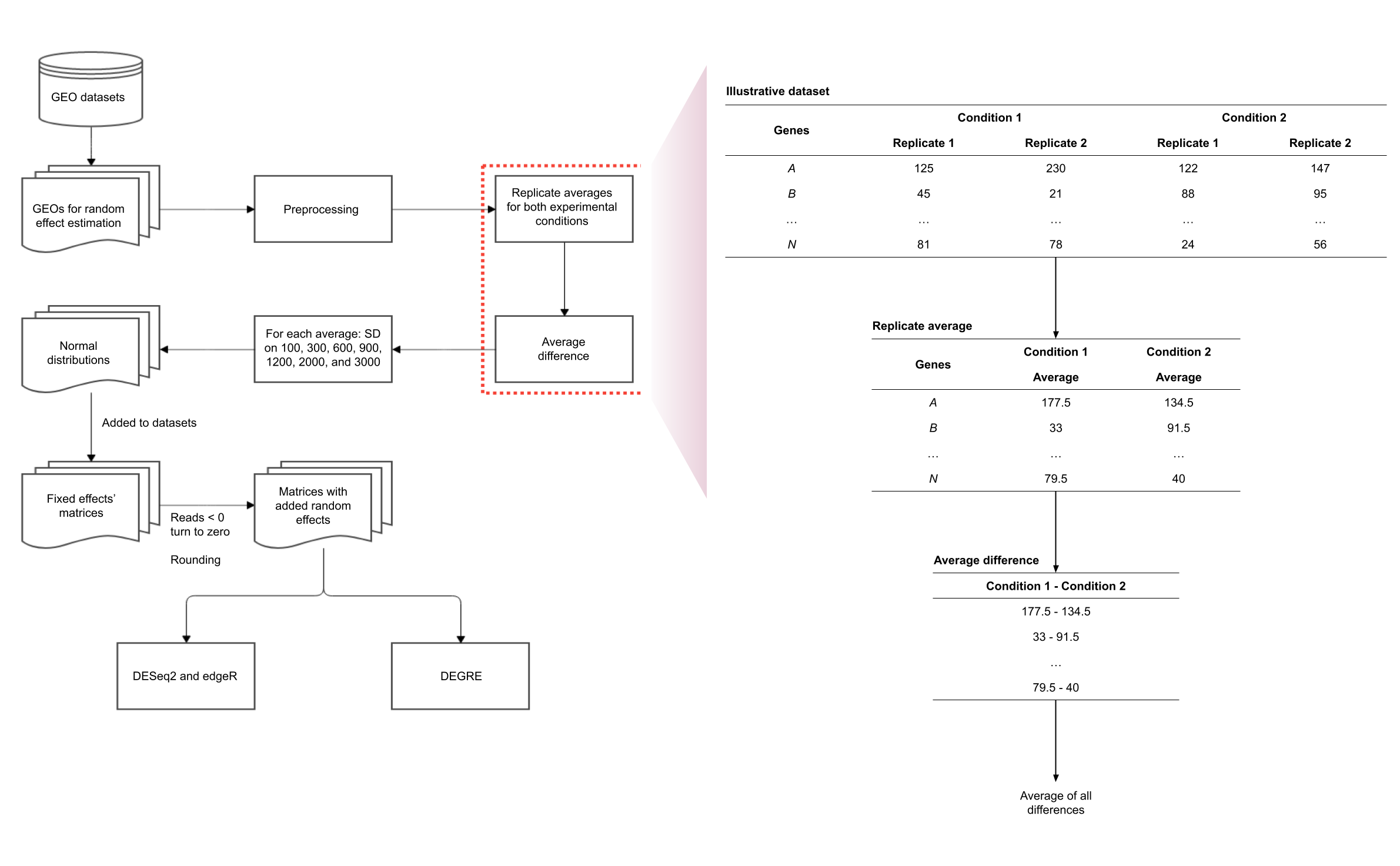

Supplement: Supplemental Information 1 — Public raw data are directed to preprocessing followed by replicate averages for the two experimental conditions and the average difference is calculated between them. For each average, the chosen set of standard deviations is used to generate normal distribution values to be added to the fixed effects’ matrices. Negative values are replaced by zero and the whole matrix values are rounded to the nearest integer. In this final step, the generated matrices with fixed and random effects are the input for DESeq2, edgeR and DEGRE. [file peerj-11-15145-s001.png]

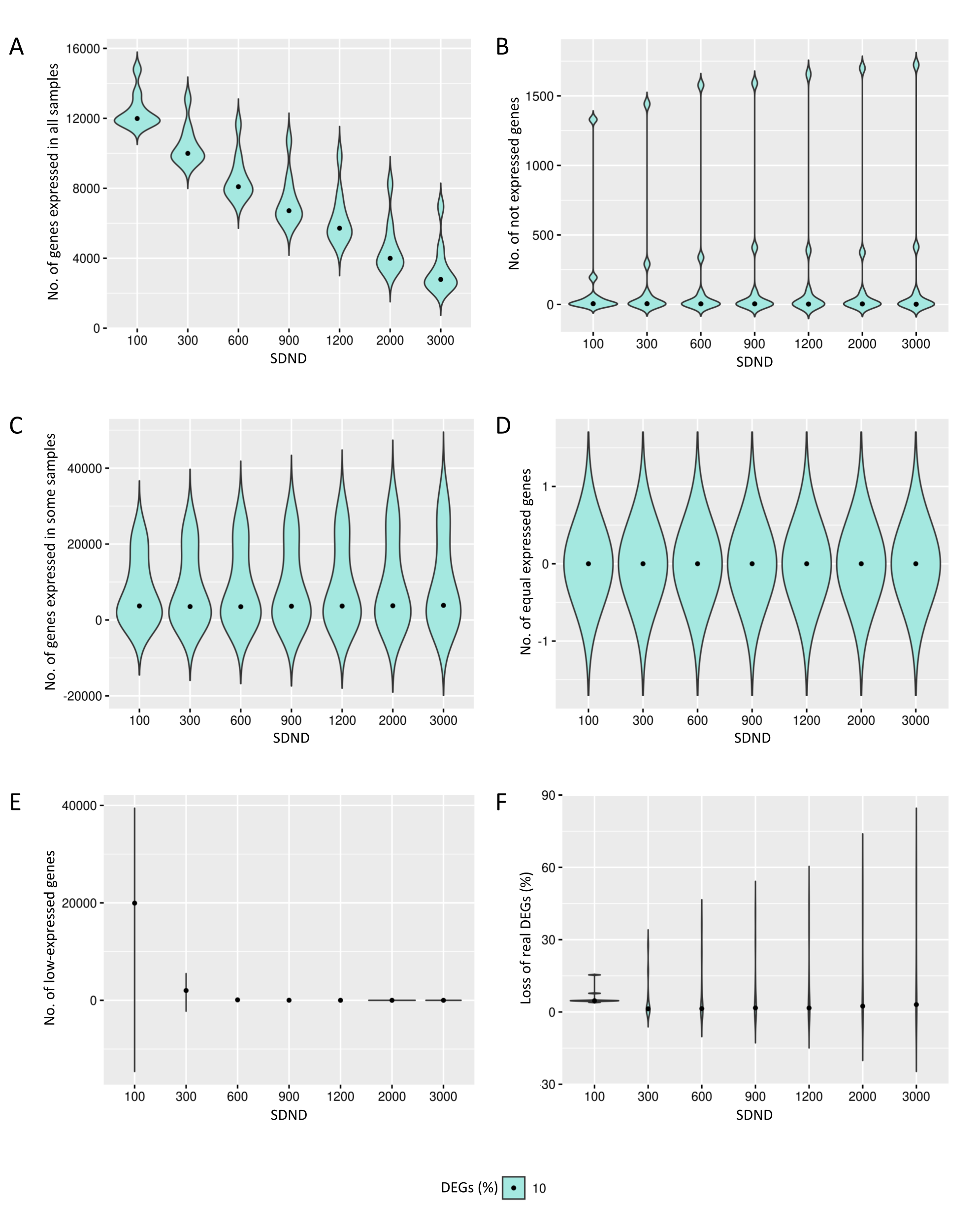

Supplement: Supplemental Information 2 — The number of genes in matrices with fixed and random effects varied from (A) expressed in all samples, (B) not expressed, (C) expressed in some samples, (D) genes with equivalent expression across samples, (E) genes with low-expression, and (F) loss of DEGs (%). [file peerj-11-15145-s002.png]

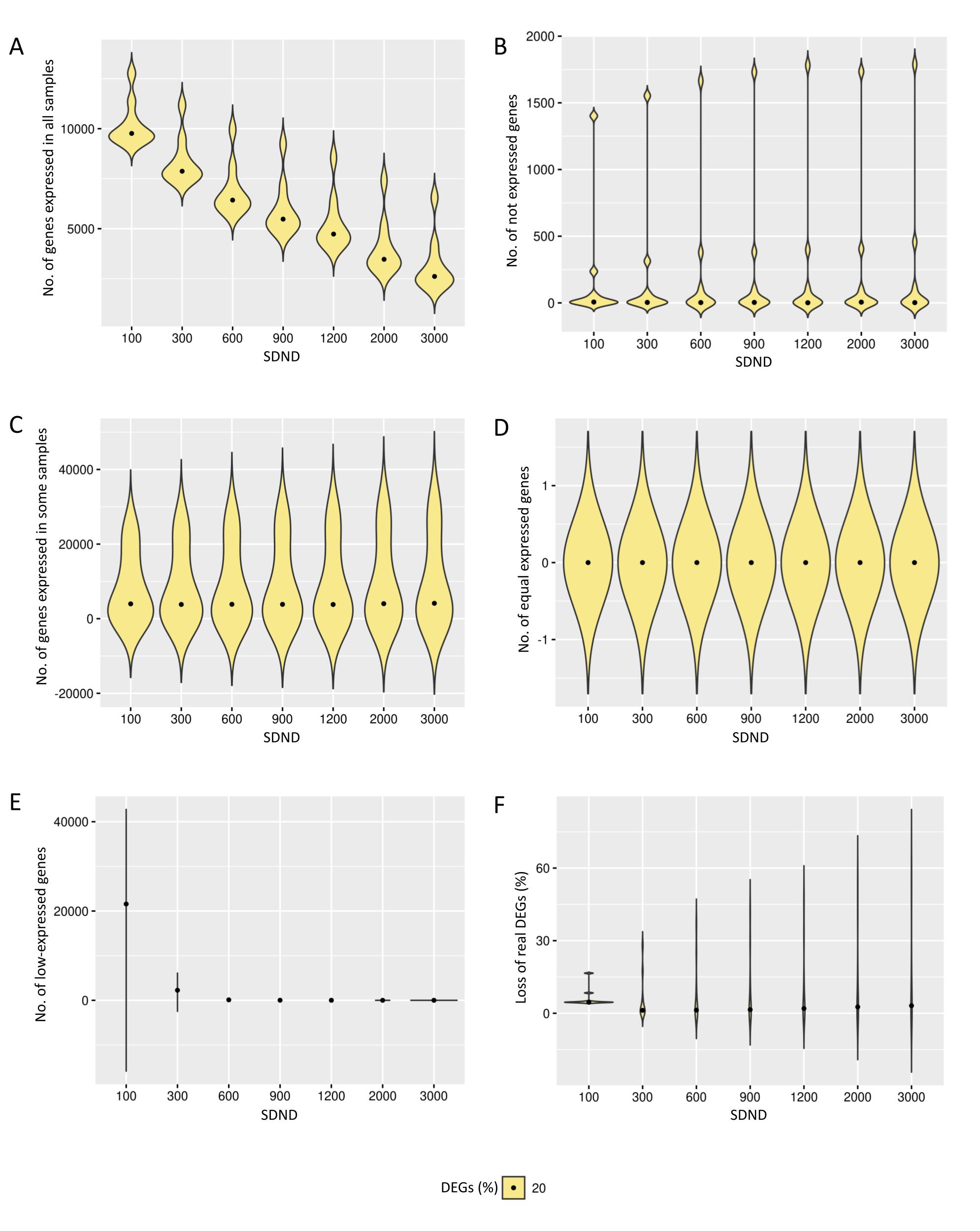

Supplement: Supplemental Information 3 — The number of genes in matrices with fixed and random effects varied from (A) expressed in all samples, (B) not expressed, (C) expressed in some samples, (D) genes with equivalent expression across samples, (E) genes with low-expression, and (F) loss of DEGs (%). [file peerj-11-15145-s003.png]

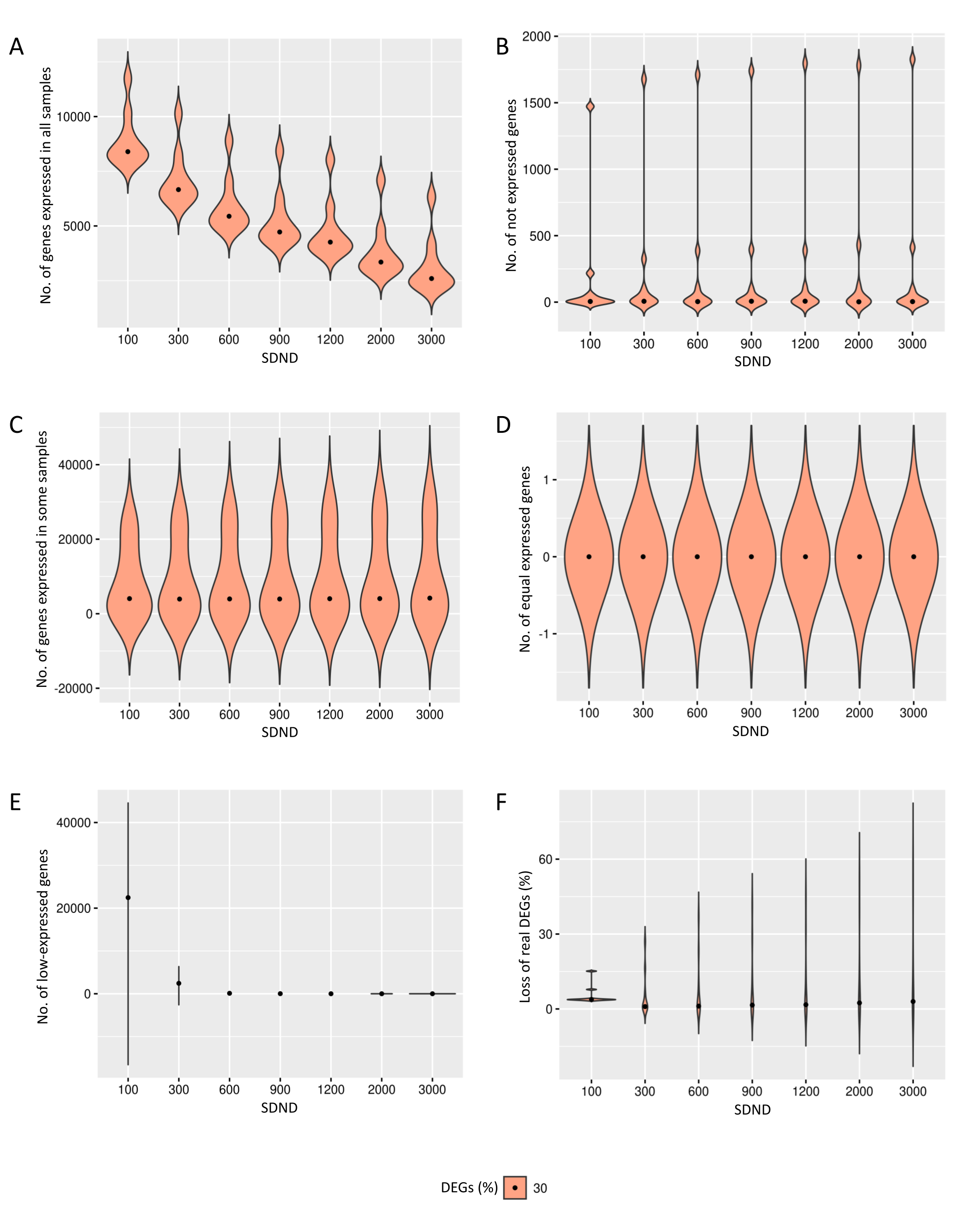

Supplement: Supplemental Information 4 — The number of genes in matrices with fixed and random effects varied from (A) expressed in all samples, (B) not expressed, (C) expressed in some samples, (D) genes with equivalent expression across samples, (E) genes with low-expression, and (F) loss of DEGs (%). [file peerj-11-15145-s004.png]

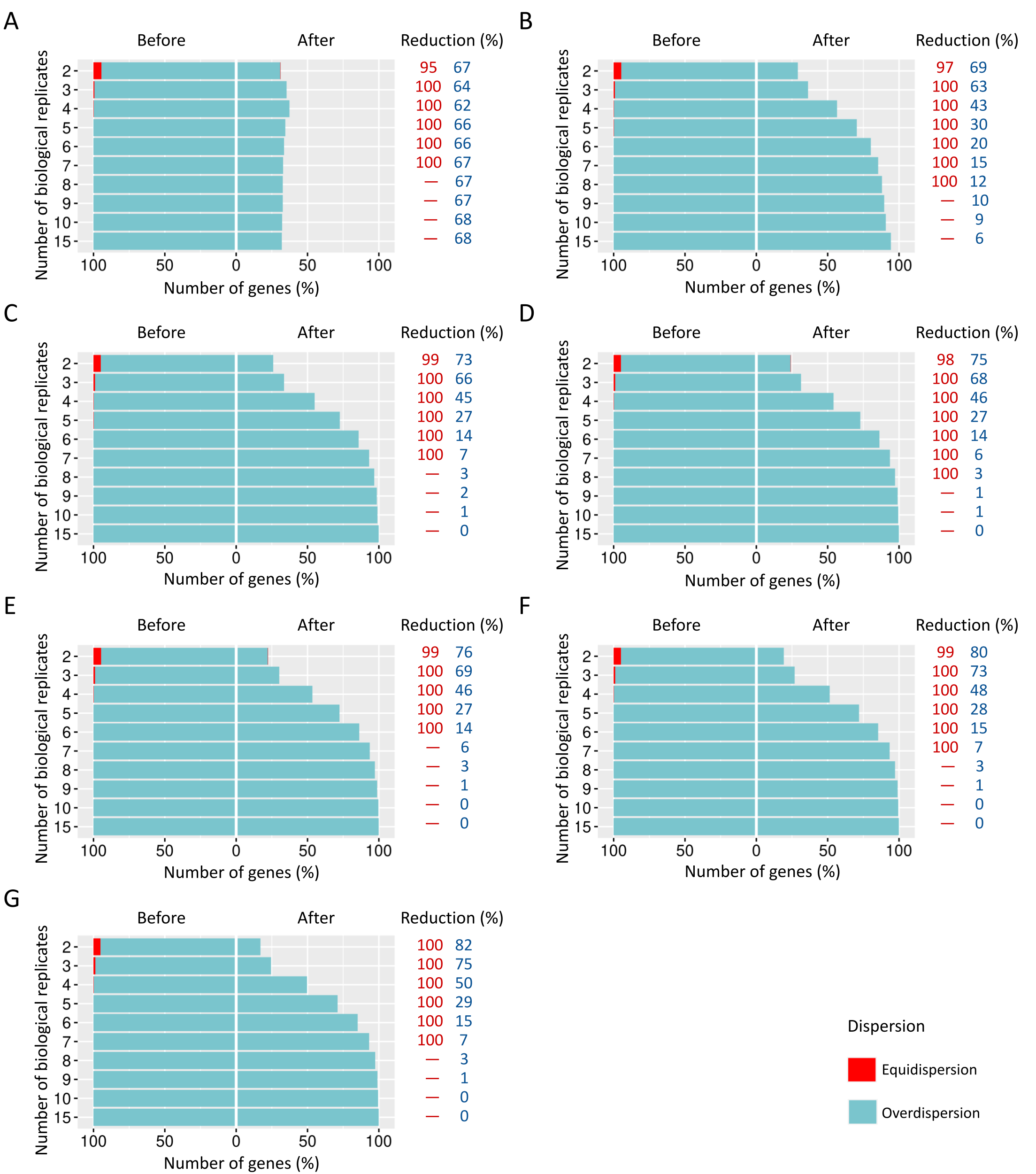

Supplement: Supplemental Information 5 — The SDNDs vary between (A) 100, (B) 300, (C) 600, (D) 900, (E) 1200, (F) 2000, and (G) 3000. The reduction percentage for each result refers to the number of equidispersed and overdispersed genes before and after the preprocessing application in matrices with fixed and random effects. [file peerj-11-15145-s005.png]

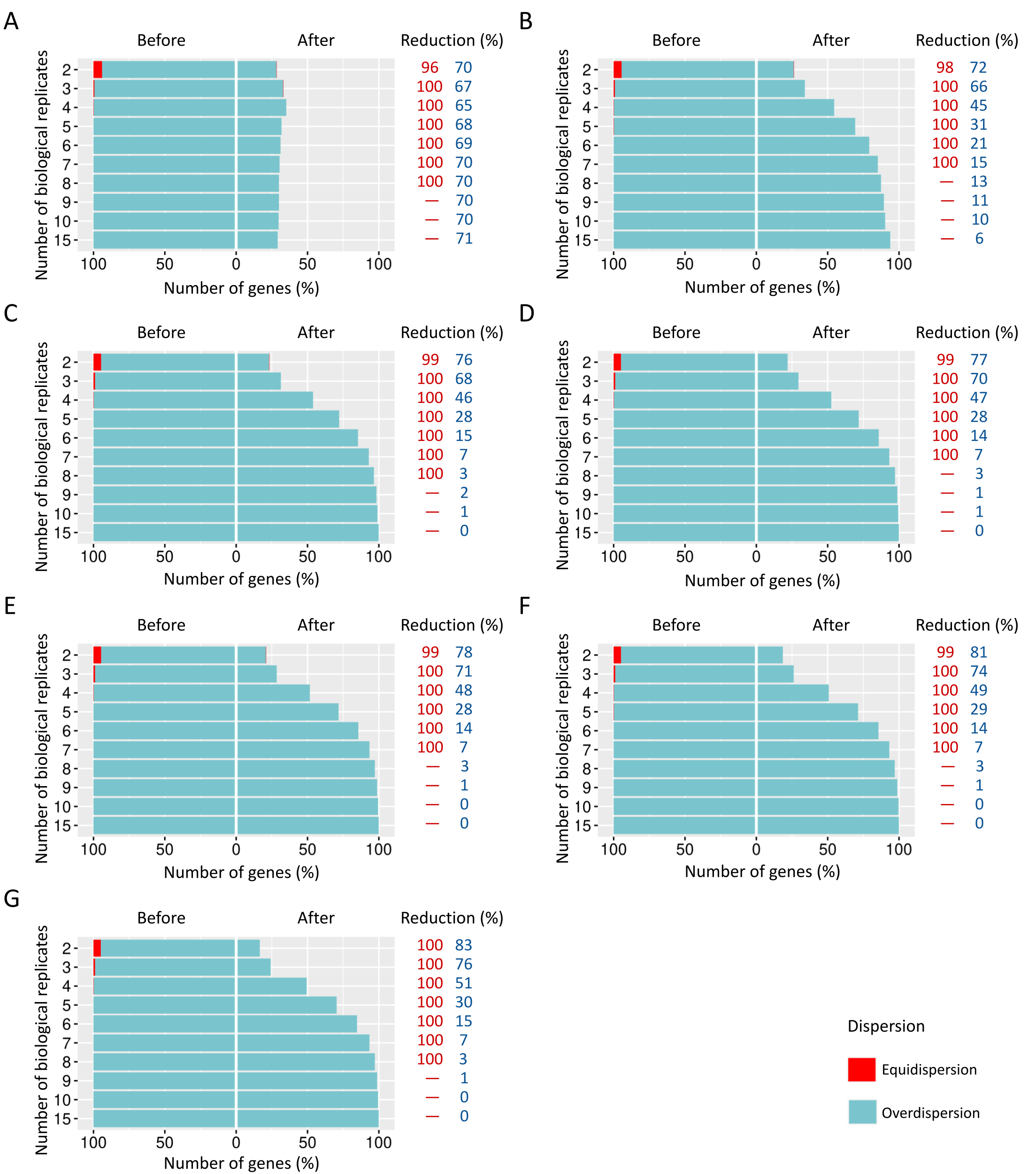

Supplement: Supplemental Information 6 — The SDNDs vary between (A) 100, (B) 300, (C) 600, (D) 900, (E) 1200, (F) 2000, and (G) 3000. The reduction percentage for each result refers to the number of equidispersed and overdispersed genes before and after the preprocessing application in matrices with fixed and random effects. [file peerj-11-15145-s006.png]
